# Dissecting the Regulation of Arachidonic Acid Metabolites by *Uncaria rhynchophylla* (Miq). Miq. in Spontaneously Hypertensive Rats and the Predictive Target sEH in the Anti-Hypertensive Effect Based on Metabolomics and Molecular Docking

**DOI:** 10.3389/fphar.2022.909631

**Published:** 2022-05-30

**Authors:** Lei Gao, Xinqin Kong, Wenyong Wu, Zijin Feng, Haijuan Zhi, Zijia Zhang, Huali Long, Min Lei, Jinjun Hou, Wanying Wu, De-an Guo

**Affiliations:** ^1^ National Engineering Laboratory for TCM Standardization Technology, Shanghai Institute of Materia Medica, Chinese Academy of Sciences, Shanghai, China; ^2^ University of Chinese Academy of Sciences, Beijing, China; ^3^ School of Chinese Materia Medica, Nanjing University of Chinese Medicine, Nanjing, China

**Keywords:** *Uncaria*, anti-hypertensive, arachidonic acid metabolites, sEH = soluble epoxide hydrolase, EETS, metabolomics, molecular docking, indole alkaloids

## Abstract

*Uncaria* *rhynchophylla* (Miq). Miq. (UR), as a traditional Chinese medicine, was employed in treating hypertension as a safe and effective therapy. The pharmacological properties of UR have characteristics of multiple biological targets and multiple functional pathways. Hypertension is related to impaired metabolic homeostasis and is especially associated with the abnormal regulation of arachidonic acid metabolites, the classical cardiovascular active compounds. This study aimed to examine the anti-hypertensive effect of UR extract (URE) and its regulating role in differential metabolic pathways. The results showed that daily administration of URE at a dose of 4 g crude drug/kg orally could exert hypotensive effects on spontaneously hypertensive rats (SHRs) for 8 weeks. Non-targeted metabolomics analysis of the plasma samples suggested that the anti-hypertension effect of URE in SHRs was associated with the reorganization of the perturbed metabolic network, such as the pathways of glycerophospholipid metabolism, linoleic acid metabolism, and arachidonic acid metabolism. For the targeted metabolomics, twenty-eight arachidonic acid metabolites in SHRs were quantitatively analyzed for the first time based on ultra-high performance liquid chromatography-tandem mass spectrometry method after URE administration. URE restored the functions of these cardiovascular active compounds and rebalanced the dynamics of arachidonic acid metabolic flux. Among them, the inhibition of soluble epoxide hydrolase (sEH) enzyme activity and up-regulation of vasodilators epoxyeicosatrienoic acids (EETs) were identified as contributors to the anti-hypertension effect of URE on SHRs, and sEH represented an attractive and promising drug-binding target of URE. With the molecular docking approach, 13 potential anti-hypertension ingredients as well as sEH inhibitors were discovered, which were worthy of further investigation and verification in future studies.

## 1 Introduction

*U. rhynchophylla* (Miq.) Miq. (UR), a traditional Chinese medicine (TCM), has been used for centuries to treat cardiovascular diseases, especially hypertension. The extracted chemical components from *Uncaria* mainly include indole alkaloids, which have been demonstrated as the bioactive constituents (Qin et al., 2021). Recent research indicates that UR extract (URE) also has other pharmacological effects, including sedation, anti-Alzheimer’s disease, anti-drug addiction (Liang et al., 2020).

Hypertension is a prevalent chronic disease and a major risk factor for cardiovascular-related morbidity and mortality (Forouzanfar et al., 2017). Since the pathogenesis of hypertension is often associated with dyslipidemia, inflammation, and oxidative stress, hypertension is considered to be a metabolic disorder (Yang and Lao, 2019). Clinical studies have established an association between serum metabolite profiles and blood pressure in hypertensive patients (Arnett and Claas, 2018; Nikolic et al., 2014) and thus the use of metabolomic approaches and techniques in hypertension has attracted increasing interest in biomarker discovery and disease diagnoses. Metabolomic studies are being used to identify small metabolites that can be considered potential biomarkers of inflammation, oxidative stress, and other cardiovascular health conditions (Currie and Delles, 2017). In clinical studies and animal models, particularly in spontaneously hypertensive rats (SHRs), changes in several metabolites were closely associated with hypertension (Aa et al., 2010; Akira et al., 2012; Akira et al., 2013).

Recent studies showed that arachidonic acid (AA) and its metabolites have a crucial role in the pathobiology of hypertension (Zhou et al., 2021). AA is an essential fatty acid that is metabolized by cyclooxygenase (COX), cytochrome P450 (CYP) enzymes, and lipid oxygenase (LOX) pathways to regulate cardiovascular function. The CYP metabolites epoxyeicosatrienoic acids (EETs) are metabolized by soluble epoxide hydrolase (sEH) to the corresponding dihydroxyeicosatrienoic acids (Das, 2018). EETs are potent endothelium-derived vasodilators, particularly in the coronaries. Enhancing the synthesis of vascular EETs or inhibiting the degradation of the EETs may represent a new therapeutic approach for cardiovascular diseases (Campbell et al., 2017). Inhibition of sEH lowered blood pressure in SHRs (Jung et al., 2005; Huang et al., 2007; Xiao et al., 2010), and sEH inhibitors are also under clinical trials for treating hypertension (Yang et al., 2017).

Previous studies have shown that *Uncaria* extracts could effectively lower systolic blood pressure (SBP) in SHRs and modulate the metabolic profiles. Ana Liu et al. found the anti-hypertensive effects were associated with lipid metabolism (dihydroceramide, ceramide, PC, LysoPC, and TXA_2_), vitamin and amino acids metabolism (nicotinamide riboside, 5-HTP) (Liu et al., 2018). Feng et al., (2019) identified six biomarkers in the urine over a 6-months treatment period. Despite these findings, the detailed mechanism underlying the anti-hypertensive effect of URE is unclear, and the regulation of arachidonic acid metabolites by URE has not been previously studied, which restricts more application of UR.

This study explored the anti-hypertension effect and underlying pharmacological mechanisms of URE in an SHR rat model. Initially, the effects of URE on the metabolic network of the plasma were analyzed using non-targeted metabolomics. AA metabolites, significantly altered after URE treatment, were then quantified using the ultra-high performance liquid chromatography-tandem mass spectrometry (UHPLC-MS) method to further investigate their regulation. Finally, molecular docking was used to elucidate mechanisms of action and discover the potential active compounds.

## 2 Materials and Methods

### 2.1 Chemicals and Reagents

Plant material of UR was kindly provided by Dr. Huiqin Pan. The voucher specimens were deposited at the herbarium of the Shanghai Institute of Materia Medica. The authentication was based on the botanical traits recorded in the Chinese Flora (http://frps.iplant.cn/frps/Uncaria). 1 kg of UR samples was ground and passed through the No. 2 sieve (850 μm), with a passing rate of more than 80%. The powder was extracted twice by refluxing with boiling water (1:15, w/v) for 20 min each time. The combined solution obtained was concentrated under reduced pressure and lyophilized using a freezing vacuum dryer. The extract was analyzed using LC-HRMS to establish the compounds present. The corresponding chromatographic fingerprinting was published in a previous study of our lab since the same preparation method was used (Feng et al., 2019).

19-hydroxyeicosatetraenoic acid (19-HETE), 20-hydroxyeicosatetraenoic acid (20-HETE), 18-hydroxyeicosatetraenoic acid (18-HETE), 17-hydroxyeicosatetraenoic acid (17-HETE), 16-hydroxyeicosatetraenoic acid (16-HETE), 11-hydroxyeicosatetraenoic acid (11-HETE), 12-hydroxyeicosatetraenoic acid (12-HETE), 9-hydroxyeicosatetraenoic acid (9-HETE), 8-hydroxyeicosatetraenoic acid (8-HETE), 5-hydroxyeicosatetraenoic acid (5-HETE), 15-hydroxyeicosatetraenoic acid (15-HETE), thromboxane-B2 (TXB_2_), prostaglandin F2α (PGF_2_α), prostaglandin D2 (PGD_2_), prostaglandin E1 (PGE_1_), prostaglandin E2 (PGE_2_), prostaglandin I2 (PGI_2_), leukotriene-E4 (LTE_4_), leukotriene-B4 (LTB_4_), 20-carboxy-leukotriene-B4 (20- carboxy—LTB4), 6-trans-leukotriene-B4 (6-trans-LTB_4_), 11.12-epoxyeicosatrienoic acid (11.12-EET), 8.9-epoxyeicosatrienoic acid (8.9-EET), 5.6-epoxyeicosatrienoic acid (5.6-EET), 14.15-epoxyeicosatrienoic acid (14.15-EET), 11.12-dihydroxy eicosatrienoic acid (11.12-DHET), 8.9-dihydroxy eicosatrienoic acid (8.9-DHET), 5.6-dihydroxy eicosatrienoic acid (5.6-DHET), 14.15-dihydroxy eicosatrienoic acid (14.15-DHET), 11.12-dihydroxy eicosatrienoic acid-d_11_ (11.12-DHET-d_11_), 5-hydroxyeicosatetraenoic acid-d_8_ (5-HETE-d_8_), leukotriene-B_4_-d_4_ (LTB_4_-d_4_), prostaglandin E_2_-d_4_ (PGE_2_-d_4_), and thromboxane-B_2_-d_4_ (TXB_2_-d_4_) were purchased from Cayman Chemicals (Ann Arbor, MI, United States). Deuterated compounds (11.12-DHET-d_11_, 5-HETE-d_8_, LTB_4_-d_4_, and PGE_2_-d_4_) were used as internal standards (IS) for quantification.

HPLC-grade acetonitrile and isopropanol were acquired from Merck KGaA (Merck, Darmstadt, Germany) and acetic acid from Sigma-Aldrich (St. Louis, MO, Unites States) was used as the mobile phase. Formic acid and ethyl acetate were purchased from Sigma-Aldrich (St. Louis, MO, United States) and used as solvents for sample extraction. Deionized water (18.2 MΩ cm at 25°C) was prepared using a Millipore Alpha-Q water purification system (Millipore, Bedford, United States).

### 2.2 Animals and Drug Administration

The animal studies were carried out after approval of the protocol by the Animal Ethics Committee of Shanghai Institute of Materia Medica (Shanghai, China). The protocol number was 2017-10-GDA-47. Male SHRs and normotensive control Wistar Kyoto rats (WKYs) at 10 weeks old were purchased from Wei Tong Li Hua Animal Center (Beijing, China). Rats were housed individually in metabolic cages in a standard animal laboratory with a 12-h light/dark cycle. Water and standard rat chow were available *ad libitum*, and the rats were acclimated to the facilities and environment for 2 weeks before the experiments were initiated.

The SHRs were then randomly divided into three groups. The following preparations were administered to the rats based on the groups to which they were assigned: vehicle (deionized water) or agents suspended in the vehicle, including captopril (CAP) at 30 mg/kg (ig per day), URE at 4.00 g/kg (measured as the quantity of crude material administered ig per day, the dose was calculated as human dose 12 g/60 kg/day). The Wistar rats were defined as the normotensive control group and given the same intervention as the SHRs group. All animals were given the gastric infusion once at the same time of day for a continuous period of 8 weeks.

### 2.3 Measurement of Blood Pressure and Heart Rate

Blood pressure was measured using a rat non-invasive tail artery blood pressure meter (SHANGHAI ALCOTT BIOTECH CO., LTD., China). The instrument was turned on and preheated for 20 min before measurement, and the pressure signal was calibrated. The rat was fixed in the fixation box, and the rat’s tail was inserted into the tail cannula to access the pulse receptor. Turn on the heating blanket under the rat and start to detect the change of pulse wave. After a stable pulse wave appeared, the blood pressure of the rat was measured by clicking the measurement button. The sphygmomanometer could record the systolic and diastolic blood pressure and heart rate of the rat simultaneously.

After the rats were acclimatized in the animal room for 1 week, all rats were trained to measure blood pressure to minimize interference with the measurement data during normal blood pressure measurement due to rat discomfort. Five sets of valid data were measured for each rat, and the average of the remaining data after excluding the highest and lowest values was taken as the result of the current measurement. All rats were measured once before dosing and used as a basis for grouping.

### 2.4 Sample Preparation

Under isoflurane anesthesia, blood was collected from abdominal aorta in sodium heparin anticoagulation tubes until rat death. Each blood sample was centrifuged at 4,000 rpm for 10 min at 4°C. The supernatant was transferred into clean 1.5 ml centrifuge tubes and frozen at −80°C until UHPLC/MS analysis was performed.

To a 300 μl aliquot of each sample, 1.5 μl of IS solution-(LTB_4_-d_4_, PGE_2_-d_4_, 11.12-DHET-d_11_, 5-HETE-d_8_ and TXB_2_-d_4_ at 500 ng/ml) and 900 µl of ethyl acetate (containing 0.025% formic acid) were added, and the samples were vortexed by vortex-mixing for 3 min. After centrifugation (4°C, 4,000 rpm) for 10 min, the 800 μl of supernatants were pooled and evaporated under nitrogen. The residue was reconstituted with methanol: water 50 μl (1:1, v/v) and 5 µl of the supernatant was injected into the UPLC-QQQ-MS system for analysis.

### 2.5 Chromatography and Mass Spectrometry Conditions

Chromatographic separation was performed on Agilent 6495 Triple Quadrupole (QQQ) LC/MS system with Agilent Jet Stream Technology (AJST) enhanced electrospray source (UHPLC-QQQ-MS) (Agilent Technologies, United States). The analytical column was a Waters ACQULITY UPLC BEH C18 (1.7 μm, 2.1 × 100 mm). The mobile phase was comprised of solvent A: 100% H_2_O (0.1% acetic acid) and solvent B: 10% isopropanol +90% acetonitrile, with gradient elution as follows: 90%–65% A at 0–3.5 min, 65%–60% A at 3.5–5.5 min, 60%–58% A at 5.5–7 min, 58%–50% A at 7–9 min, 50%–35% A at 9–15 min, 35%–25% A at 15–17 min, 25%–15% A at 17–18.5 min, 15%–5% A at 18.5–19.5 min, 5% A at 19.5–21 min, 5%–90% A at 21–22.5 min, and 90% A at 22.5–27 min. The flow rate was kept at 0.30 ml/min. The temperatures of the autosampler and column were kept at 4 and 40°C, respectively. The injection volume of all samples was set at 5.0 μl.

The mass spectrometric analysis was done in the negative ion mode (AJST-ESI^-^). The product ions and optimal collision energies of all the compounds were selected with MassHunter Optimizer software (B.09). The ESI source parameters were optimized with MassHunter Source and iFunnel Optimizer software (B.09). The AJST-ESI^-^ conditions were finally set as follows: capillary voltage, 3,500 V; drying gas flow 14 L/min at a temperature of 200°C; nebulizer gas flow 35 psi. All the compounds were detected in dynamic multiple reaction monitoring (MRM) mode with optimized transitions and collision energies. Samples were analyzed randomly for unbiased measurement with a deuterated reference solution as internal standards to ensure accuracy and reproducibility.

### 2.6 Data Processing and Pattern Recognition Analysis

The plasma samples were analyzed and manipulated using MassHunter software (Agilent Technologies, Inc., United States). MassHunter Optimizer 2.0 optimization software was used to automatically optimize mass spectrometry parameters, ion pairs, and collision voltages for each analyte to be analyzed. Qualitative data were analyzed using QQQ Qualitative analysis (version B.06.00., Agilent Technologies, Inc., Unitd States); quantitative data were analyzed using QQQ Quantitative Analysis (version B.03.02., Agilent Technologies, Inc., United States). Before chemometric analysis, the missing values for each sample class were treated using the 80% rule. The data were introduced to SIMCA-P V14.0 (Umetrics, Sweden, Stockholm) for orthogonal partial least squares discriminant analysis (OPLS-DA). The quality of the models was evaluated with the relevant R^2^ and Q^2^. Statistical significance of blood pressure, heart rate and plasma samples were analyzed using one-way analysis of variance (ANOVA) with the Tukey HSD test for post hoc analysis, implemented in GraphPad Prism 9.0 (https://www.graphpad.com/scientific-software/prism/).

### 2.7 Identification of Differential Endogenous Metabolites and Metabolic Pathway Analysis

For identification of differential endogenous metabolites, the accurate MS fragments with the metabolites were searched in One-MAP (One-step Metabolomics, Dalian ChemDataSolution Information Technology Co. Ltd., Dalian, China) and online free databases such as the Human Metabolome Database (http://www.hmdb.ca/), Metlin (http://metlin.scripps.edu/), and KEGG (http://www.genome.jp/kegg/pathway.html). Pathway analysis was performed by MetaboAnalyst 5.0 (https://www.metaboanalyst.ca/), which is a useful web-based tool for pathway analysis and visualization of metabolomics.

### 2.8 Molecular Docking

Simulation of the binding of alkaloid monomers to the metabolic enzyme sEH of EETs was carried out based on the molecular docking software SwissDock. Firstly, the structural formulae of corynoxine, corynoxine B, geissoschizine methylether, uncarine B, isomitraphylline, isorhynchophylline, rhynchophylline, isocorynoxeine, mitraphylline, hirsutine, corynoxeine, hirsuteine, and uncarine A were drawn using Chemdraw 3D software to establish the 3D structures of the ligands, which were energy-optimized and saved in. mol2 format for subsequent docking analysis. Secondly, the X-ray crystal structures of sEH were downloaded from the RCSB Protein Data. The X-ray crystal structure of sEH (PDB No. 1EK2) was downloaded from the RCSB Protein Data Bank (http://www.rcsb.org) at a resolution of 3 Å. The X-ray crystal structure was analyzed to identify reasonable binding sites and binding pockets that could be used to define the target site and determine the rationality of the docking conformation. SwissDock was used for fast docking to generate different conformational orientations and to obtain electrostatic and van der Waals interactions between the ligand molecule and the binding site, from which the FullFitness (kcal/mol) and Estimated ΔG (kcal/ mol) scores and the 3D conformations of the corresponding protein-small-molecule docking complexes are shown. Finally, the mapping was analyzed using LigPlus.

## 3 Results and Discussion

### 3.1 The Effect of URE on Blood Pressure and Heart Rate

In this experiment, systolic blood pressure (SBP), diastolic blood pressure (DBP), and heart rate (HR) were measured to assess the anti-hypertension effect of URE over 8 weeks of intragastric administration. In contrast to blood pressure measurements under anesthesia, the non-invasive tail artery sphygmomanometry used in this study, performed in a conscious state of the rats, was able to exclude changes in blood pressure caused by anesthetic drugs.

**FIGURE 1:**
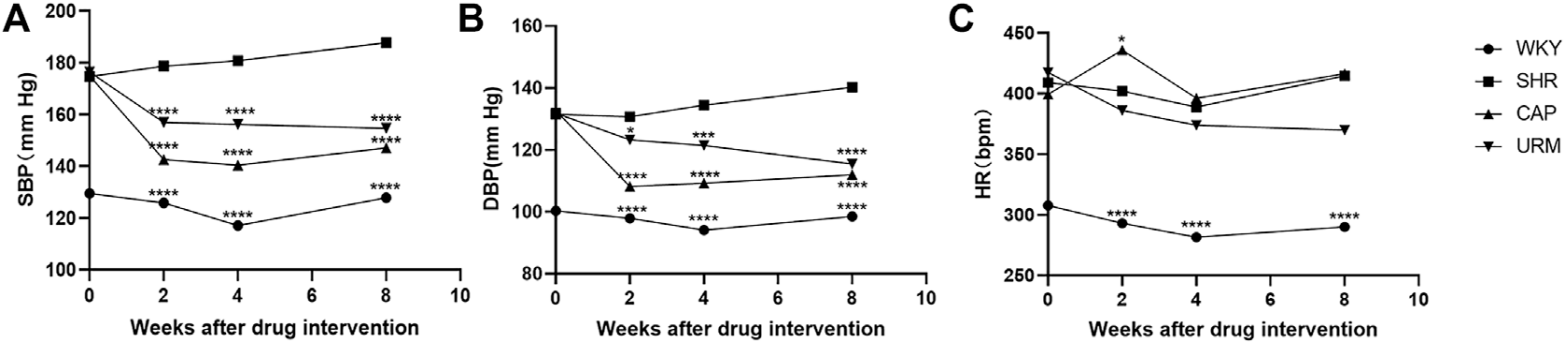
Effect of URE on blood pressure and heart rate in spontaneously hypertensive rats. **(A)** SBP, **(B)** DBP, **(C)** HR. Values are mean ± SD (*n* = 10). ∗*p* < 0.05, ∗∗*p* < 0.01, ∗∗∗*p* < 0.001, ∗∗∗∗*p* < 0.0001 vs. SHR (one-way ANOVA with the Tukey HSD test). WKY, control group; SHR, model group; CAP, positive control group; URE, treatment group.

The effects of long-term URE treatment on blood pressure and HR in SHRs were shown in Figure 1. The SBP, DBP, and HR of the control group (WKY) remained stable during the 8-weeks time course. The SBP and DBP in the model group (SHR) increased slowly throughout the experiment and eventually remained above 180 and 140 mmHg, a typical trend of blood pressure in SHRs - a slow increment in blood pressure with the age increase. Blood pressure and HR were measured at the second, fourth, and eighth weeks after treatment with CAP and URE, respectively. The SBP and DBP in the CAP and URE groups decreased rapidly after 2 weeks of administration and then remained relatively stable. After 8 weeks of treatment, the SBP and DBP of the CAP and URE groups were significantly lower than those of the SHR model group (*p* < 0.0001). In conclusion, in terms of the effects on SBP and DBP, the URE group showed the same trend as the CAP, and there was no significant difference between the two groups.

As for the effects on HR, the CAP group fluctuated dramatically and was the same as that of the model group at the end of 8 weeks of administration, whereas the URE group decreased slowly during the dosing period. This phenomenon may, to some extent, indicate the difference in the anti-hypertensive mechanism between URE and CAP. CAP was an angiotensin-converting enzyme inhibitor (ACEI), which reduced blood pressure without reflex tachycardia and compensatory change in heart rate. The perturbation of heart rate by URE might be attributed to its characteristics of multiple biological targets and functional pathways, but this effect was not significant and more data were needed in future studies.

### 3.2 Non-Targeted Metabolomics Pattern Analysis

**FIGURE 2:**
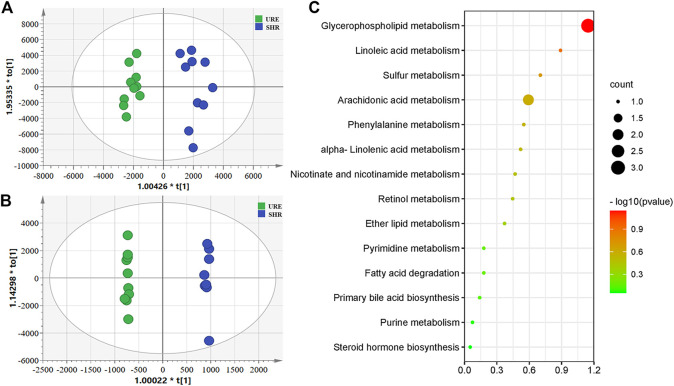
**(A and B)** showed the OPLS-DA scores based on metabolic profiling of the URM and SHR groups, and **(C)** was the summary of differentially metabolic pathway analysis with MetaboAnalyst. **(A)** Scores plot from OPLS-DA model classifying URE and SHR groups under the positive ion mode; **(B)** Scores plot from OPLS-DA model classifying URE and SHR groups under the negative ion mode; **(C)** The differential metabolite enrichment dot bubble maps with the data of count.

To obtain the global profile of metabolites in the plasma of different experimental groups, the non-targeted metabolomics analysis was performed with UHPLC-MS under optimized conditions. The OPLS-DA, a supervised method to classify groups and extract potential biomarkers, was subsequently used to investigate the specific compounds altered by the URE treatment. The corresponding score plots from the OPLS-DA showed apparent separation between the SHR and URE groups in both positive and negative ion modes, indicating a differential metabolite composition between the two groups (Figures 2A,B. In addition, the OPLS-DA model parameters, including R2(X), R2(Y), and Q2 had values of 0.785, 0.944, and 0.468 in the positive ion mode, and 0.784, 0.999, and 0.825 in the negative ion mode, exhibiting good reproducibility and predictability in explaining the differences between the two groups.

Variable importance in projection (VIP) is the variable weight value of the OPLS-DA model variables and can be used to detect biologically significant differential metabolites. Fold Change (FC) is usually used to calculate the difference in expression of a metabolite between two groups. Moreover, statistical analysis is performed using a *t*-test to assess significant differences between the two groups. Based on VIP > 1, FC > 2, and *p* < 0.05, 573 and 539 potential differential metabolites were screened from the positive and negative ions, respectively. For further annotation, the MS^1^ and MS^2^ spectrum of metabolites were further analyzed in One-MAP (One-step Metabolomics, Dalian ChemDataSolution Information Technology Co. Ltd., Dalian, China). 78 and 27 metabolites were identified in the positive and negative ion modes by the MS/MS fragments, including lipids and lipid-like molecules, organic acids and derivatives, amino acids and derivatives, hormones, vitamins, and other compounds. For an in-depth understanding of the defined differential metabolites, metabolic changes were investigated based on pathway analysis. Using MetaboAnalyst to search for differential metabolite-related pathways, there were significant changes in 14 metabolic pathways. The results showed that the regulation from URE was mainly related to the pathways of glycerophospholipid metabolism, linoleic acid metabolism, sulfur metabolism, and arachidonic acid metabolism, some of which were strongly associated with hypertension (Figure 2C).

Several metabolomic results of hypertension suggested that specific metabolites involved in the glycerophospholipid pathway had an important role in nitric oxide production as well as vascular remodeling (Jiang et al., 2015; Chu et al., 2016; Ke et al., 2018) and were even associated with the development of ischaemic hypertensive stroke (Guo et al., 2019). Linoleic acid could increase oxidative and inflammatory stresses, and abnormalities in linoleic acid metabolism was a risk factor for the development of hypertension by directly or indirectly influencing the cardiovascular disease process (Sato et al., 2005; Feig et al., 2008) as evidenced by relevant clinical studies (Feig and Johnson, 2003; Sun et al., 2021). Selhorst et al., (2000) found that idiopathic intracranial hypertension was associated with abnormal retinol metabolism by measuring serum retinol values in normal subjects and patients with idiopathic intracranial hypertension (IIH). Bujak et al., (2016) found increased plasma levels of free fatty acids and enhanced lipolysis in patients with pulmonary arterial hypertension (PAH). In addition, metabolomic studies in SHRs highlighted that various metabolites involved in phenylalanine metabolism, nicotinamide metabolism, pyrimidine metabolism, and steroid hormone biosynthesis were also associated with the development of hypertension.

Plenty of studies have demonstrated that hypertension is correlated with disturbed substance metabolism. In the current study, the URE treatment significantly altered some metabolic perturbations, which could be a crucial mechanism of its therapeutic effect. This was different from the regulatory effect of CAP on the metabolomic profile. There were few differential metabolites screened and identified in the CAP-treatment group, and only two pathways with *p*-value < 0.05 were obtained, including glutathione metabolism and sulfur metabolism (Supplementary Figure S3). By contrast, URE restored the functions of key metabolic pathways and thus elicited metabolic network reorganization.

### 3.3 Targeted Detection of Arachidonic Acid Metabolites in Plasma Samples

The aforementioned pathway enrichment results (Figure 2C) showed that the metabolism of AA, which was closely associated with hypertension, was significantly altered before and after URE treatment. AA is an important ω-6 polyunsaturated fatty acid and a precursor of several active components in the body, which plays an important role in cardiovascular diseases (Aspromonte et al., 2014; Jamieson et al., 2017; Zhang et al., 2021). In this study, a UHPLC-MS method was developed to further investigate the regulation of AA metabolites by URE. This method was sensitive, reproducible, and allowed simultaneous analysis of a range of AA metabolites without the requirement of derivatization.

The analysis was performed on an ACQUITY UPLC BEH C18 (2.1 × 100 mm, 1.7 μm) column. In the negative ion mode (AJST-ESI^-^), AA metabolites were ionized into deprotonated ionic substances ([M-H]^-^) (Zhang et al., 2007), which were used for EETs, HETEs, PGs, LTs, etc. as precursor ions for collision-induced decomposition (CID). The dynamic MRM method was chosen for further experiments, which was more suitable for complex samples with low levels of target analytes than the traditional time-segmented MRM method. The dynamic MRM allowed monitoring of more MRM ion pairs in a single acquisition while maintaining the high sensitivity, selectivity, and reproducibility of the chromatographic results (Dong et al., 2015). Other parameters were summarized in Table 1, including retention times for the analytes and internal standards, as well as the fragmentation voltage (Fragmentor), cell accelerator voltage (CA), and collision energy (CE) of AA metabolites in dynamic MRM mode. The method has been fully validated, and the result was highly precise, accurate, and met the methodological requirements for quantifying *in vivo* biological samples. Validation details were shown in Supplementary Figure S1.

**TABLE 1:** LC-MS/MS parameters used for the analysis of AA metabolites.

Analyte	Rt (min)	Precursor ion	Product ion	Fragmentor (V)	CA (V)	CE (V)
5.6-EET	17.69	319.2	191.3	380	5	8
8.9-EET	17.50	319.2	69.21	380	5	13
11.12-EET	17.29	319.2	167.1	380	5	12
14.15-EET	16.75	319.2	301.1	380	5	8
5.6-DHET	14.05	337.2	319.1	380	5	12
8.9-DHET	13.34	337.2	127.2	380	5	24
11.12-DHET	12.79	337.2	167.1	380	5	20
14.15-DET	12.23	337.2	207.1	380	5	16
5-HETE	16.46	319.2	257.2	380	5	12
8-HETE	15.79	319.2	301.3	380	5	12
9-HETE	16.10	319.2	167.2	380	5	16
11-HETE	15.50	319.2	167.2	380	5	16
12-HETE	15.79	319.2	179.2	380	5	12
15-HETE	15.03	319.2	301.3	380	5	12
16-HETE	14.25	319.2	233.3	380	5	12
17-HETE	14.13	319.2	247.2	380	5	12
18-HETE	13.96	319.2	261.3	380	5	16
19-HETE	13.77	319.2	301.1	380	5	16
20-HETE	13.75	319.2	289.2	380	5	16
LTB4	11.21	335.2	195.1	380	5	16
20-Carboxy-LTB4	5.25	365.2	347.2	380	5	16
6-trans-LTB4	10.90	335.2	195.3	380	5	12
PGD2	7.46	351.2	271.2	380	5	16
PGE1	7.31	353.2	317.2	380	5	12
PGE2	7.14	351.2	333.2	380	5	8
PGF2α	6.87	353.2	291.2	380	5	20
PGI2	5.21	369.2	245.2	380	5	28
TXB2	6.32	369.2	169.1	380	5	20
11.12-DHET-d11	12.71	348.3	167.2	380		20
5-HETE-d8	16.36	327.3	309.2	380	5	12
LTB4-d4	11.17	339.2	197.1	380	5	16
PGE2-d4	7.11	355.2	319.1	380	5	12
TXB2-d4	6.28	373.2	173.1	380	5	16

**FIGURE 3:**
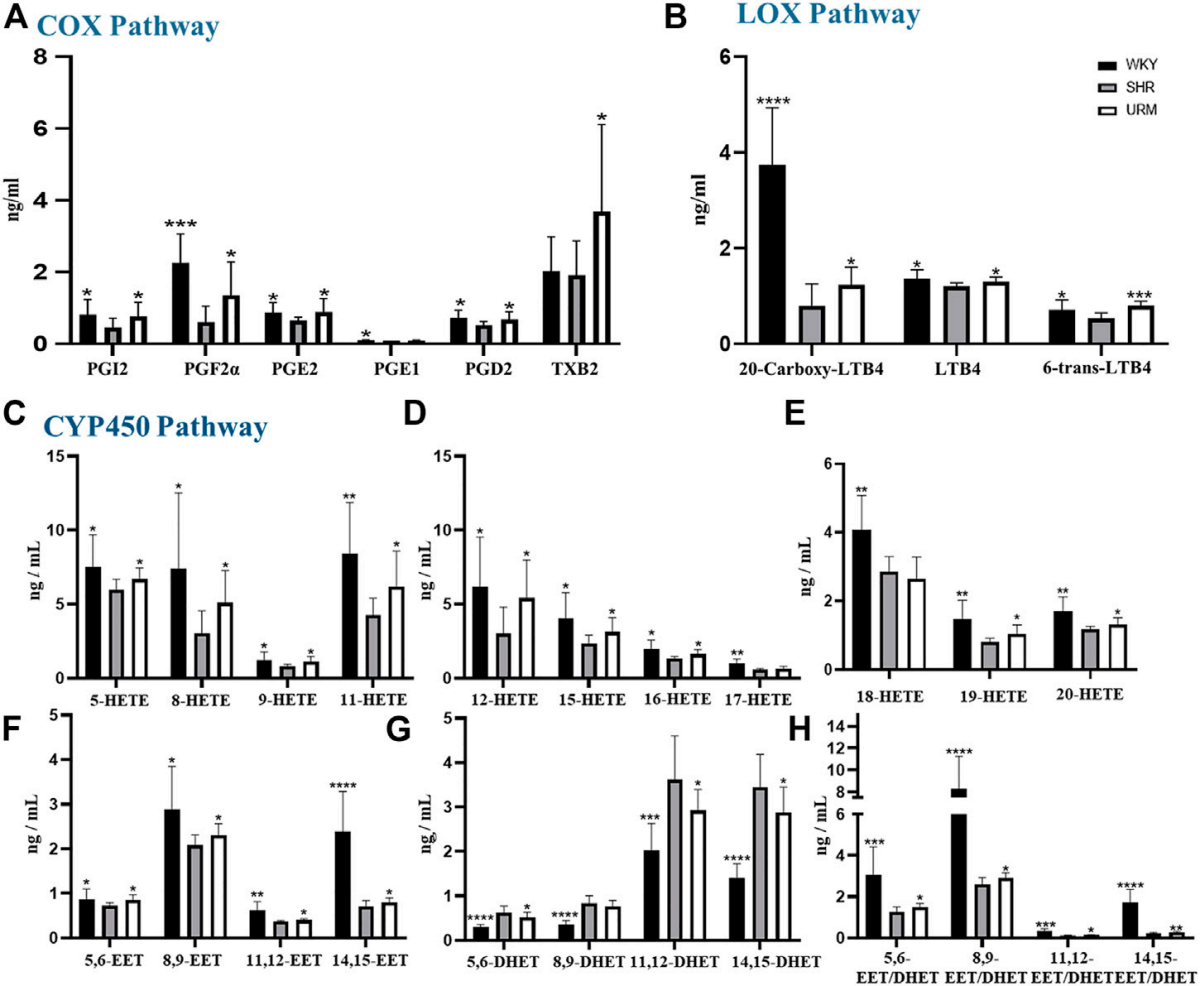
Effects of URE on arachidonic acid metabolites concentrations in plasma of spontaneously hypertensive rats. **(A)** were the concentrations of PGs, PGI2, and TXs in the plasma of different groups of rats, **(B)** were the concentrations of LTs; **(C–E)** were the concentrations of HETEs. **(F)** and **(G)** were the concentrations of EETs and DHETs, and **(H)** were the EET/DHET ratios. Values are mean ± SD (*n* = 8). ∗*p* < 0.05, ∗∗*p* < 0.01, ∗∗∗*p* < 0.001, ∗∗∗∗*p* < 0.0001 vs. SHR.

AA is metabolized by COX, LOX, and CYP450 enzymes to form several metabolites. The established LC-MS/MS method was used to detect the content of AA metabolites in plasma samples from rats in the WKY, SHR, and URE groups, and the results were shown in Figure 3.

#### 3.3.1 COX Pathway

COX-1 and COX-2 convert AA to prostaglandins, prostacyclin, and thromboxanes. Prostaglandins are unsaturated fatty acids that exert various biological activities, such as inducing vasodilation and inhibiting platelet aggregation. Many studies showed that PGE_2_ and PGD_2_ have hypotensive activity (Smyth et al., 2009; Foudi et al., 2008; Davis et al., 2004). PGI_2_ also has a similar function to prostaglandins and can be used to treat PAH (Dorris and Peebles, 2012; O'Connell et al., 2016). In contrast, thromboxane has platelet aggregation and vasoconstriction functions. As shown in Figure 3A, the plasma concentrations of PGF_2α_, PGE_1_, PGE_2_, and PGD_2_ in the SHRs were significantly reduced, but all were up-regulated after URE treatment. In addition, prostacyclin showed the same characteristics of changes as prostaglandins. As classical inflammatory factors, regulation of prostaglandins was crucial to maintain homeostasis and prevent inappropriate inflammation. Inflammatory processes were important participants in the pathophysiology of hypertension, and inflammation regulation could contribute to the development of therapeutic approaches for hypertension and its complications (Savoia and Schiffrin, 2006).

#### 3.3.2 LOX Pathway

LOXs catalyze the dioxygenation of polyunsaturated fatty acids to their corresponding hydroperoxyeicosatetraenoic acids (HPETEs), which are subsequently converted to hydroxyeicosatetraenoic acids (HETEs), leukotrienes (LTs), and lipoxins (LXs). LTs are important mediators in the development of inflammation and allergy and strongly correlated with asthma and rhinitis. Studies showed that LTB_4_ contributes to PAH (Tian et al., 2014; Li et al., 2020). In this experiment, a total of three LTs were detected, as shown in Figure 3B. Compared with the control group, the concentrations of all the three compounds in the model group were significantly decreased. They could be increased after URE administration, showing a strong correlation between LTs and hypertension. The underlying molecular mechanism of this interesting correlation was unclear and needed to be studied subsequently.

#### 3.3.3 CYP450 Pathway

CYP450 enzymes contain multiple isoforms that convert AA into different metabolites, mainly produce HETEs by ω-hydroxylase and EETs by epoxygenase. In this section, we measured various HETE components, and the results were shown in Figures 3C–E. The plasma concentrations of all the HETEs in the model group were significantly lower than those in the normal control group. Except for 17-HETE and 18-HETE, the plasma concentrations of all the other HETEs were significantly upregulated after URE treatment. As reported, 12-HETE and 15-HETE function as endothelium-derived relaxing factors (EDRFs) in arteries and contribute to vasodilation (Chawengsub et al., 2009). URE restored the functions of these vasodialating factors and rebalanced the dynamics of metabolic flux. By contrast, 20-HETE mediates contractile response through activation of Rho-kinase and sensitizes vascular SMCs to constrictors (Randriamboavonjy et al., 2003; Kizub et al., 2016). It is hypothesized that in this experiment, the changes in 20-HETE may be a mechanism of negative feedback regulation, in which sustained hypertension leads to the inhibition of 20-HETE synthesis, resulting in a decrease in its plasma concentration. After the administration of the anti-hypertensive drug, the feedback mechanism was disturbed, and the inhibition of 20-HETE synthesis was diminished so that the plasma concentration was adjusted back to the normal level.

EETs, which are metabolized by epoxygenases, have various biological effects, including anti-inflammatory, cardiovascular and metabolic disease modulation (Tacconelli and Patrignani, 2014; Lai and Chen, 2021). Their effects on hypertension are mainly vasodilative through calcium-dependent potassium channels (Campbell et al., 1996; Campbell and Falck, 2007). The plasma concentrations of EETs in SHRs were significantly reduced but regulated after URE administration (Figure 3F). Among the four isomers, 8.9-EET had the highest plasma concentration, and 11.12-EET had the lowest. 14.15-EET in the control group showed a dramatic difference from the SHR group, and significant back-regulation was also observed after URE administration. In contrast, DHETs, as inactive hydrolysis products of EETs by the sEH enzyme, showed an opposite trend in the WKY, SHR, and URE groups (Figure 3G). Furthermore, the ratio of the concentration of EETs to DHETs in rat plasma was analyzed, from which we could infer sEH activity (Stefanovski et al., 2020). The results showed that EETs/DHETs in the plasma of spontaneously hypertensive rats were significantly up-regulated after URE treatment (Figure 3H), indicating that the hydrolytic activity of the sEH enzyme was inhibited. The above results suggested that URE may exert a hypotensive effect by inhibiting sEH enzyme activity and up-regulating the concentration of EETs. The therapeutic potential for manipulating EETs through sEH inhibitors has been well recognized for the past decade. Inhibition of sEH lowered blood pressure in SHRs (Jung et al., 2005; Huang et al., 2007; Xiao et al., 2010). Moreover, the biological actions of EETs had the potential to prevent complications by improving endothelial function and decreasing organ damage in cardiovascular disease (Imig, 2012). It provided a novel therapeutic approach to cardiovascular disease.

### 3.4 Molecular Docking Analysis for Potential Active Ingredients

Targeted metabolomics results suggested that sEH enzymes might be the potential target for the hypotensive effect of URE. Therefore, we performed the target validation using the molecular docking approach.

**FIGURE 4:**
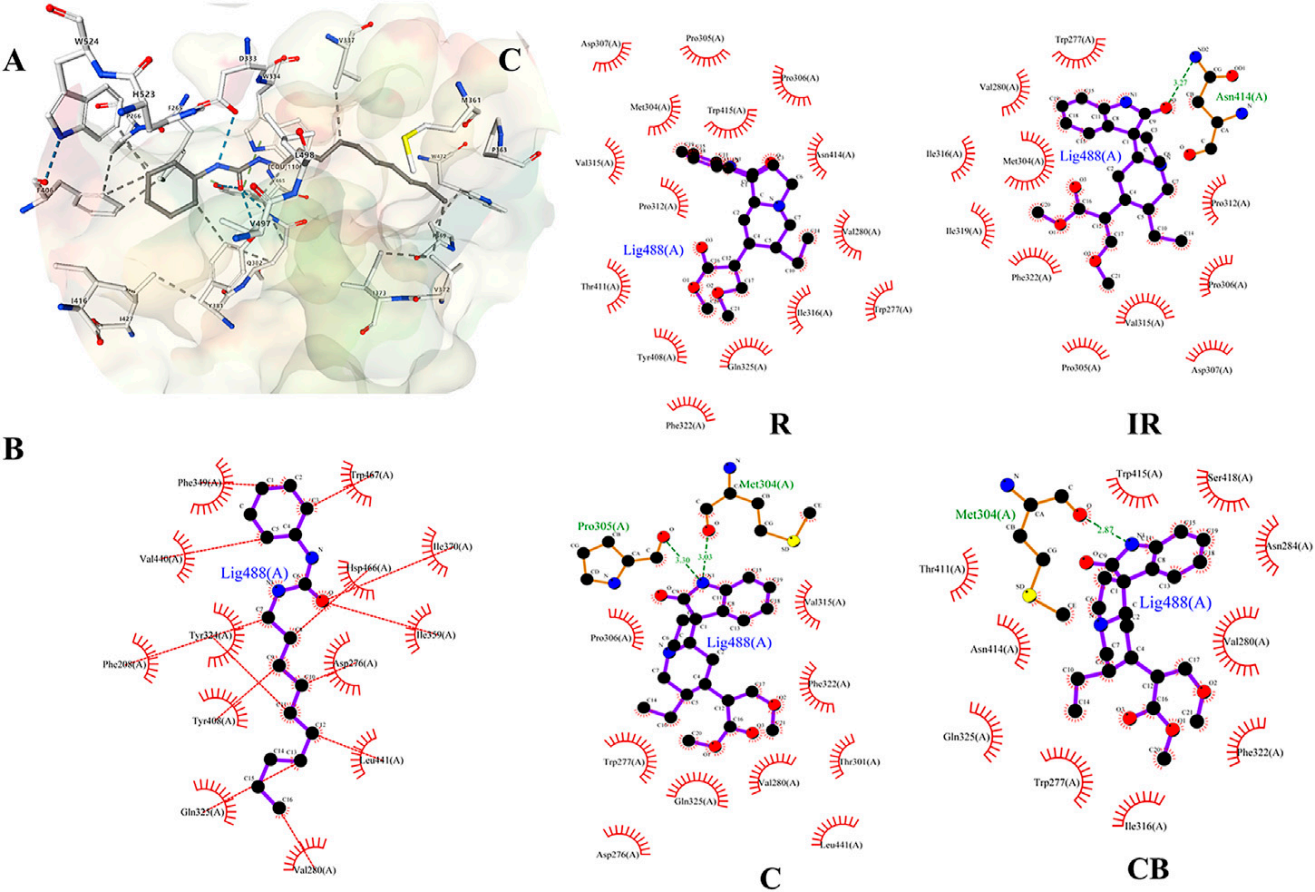
**(A)** Docking diagram of components and protein molecules. The binding pattern between sEH protein and ligand CDU; **(B and C)** were 2D-docking poses. **(B)** The binding pattern between sEH protein and ligand CDU; **(C)** The binding pattern between R, IR, C, CB and sEH protein. Black dots: carbon atoms; blue dots: nitrogen atoms; red dots: oxygen atoms; green dotted lines: hydrogen bonds; red combs: amino acid residues.

First, the protein-ligand crystal structure of murine sEH (PDB ID: 1EK2) was obtained from the PDB database. The receptor-ligand interactions were analyzed and displayed using the NGL protein web visualization tool, and the 3D visualization was shown in Figure 4A. There were multiple intermolecular interaction forces, which allowed small molecules to bind tightly to the receptor. Next, A chain was selected and analyzed using Protein-Ligand Interaction Profiler (PLIP) for key amino acid residues, intermolecular hydrophobic interactions, and hydrogen bonding interactions. The key amino acid residues were Hsp 466, Leu 441 and Tyr 408, etc. The specific results were shown in Supplementary Tables S1, S2.

The binding pattern of ligand 1-cyclohexyl-3-dodecyl urea (CDU) in sEH was predicted using the SwissDock program, and the binding conformation was ranked according to FullFitness (kcal/mol). The more tightly the protein was bound to the small molecule, the more energy was released and the lower the FullFitness (kcal/mol) and Estimated ΔG (kcal/mol). Therefore, the binding conformation of CDU and sEH with FullFitness of −2564.3965 kcal/mol and Estimated ΔG of −8.827662 kcal/mol was selected for the analysis. As shown in Figure 4B, although the binding of CDU to sEH did not form hydrogen bonding interactions, the formation of hydrophobic interactions between the carbon and oxygen atoms in the CDU and amino acid residues such as Hsp 466, Leu 441, and Tyr 408 provided strong Van der Waals forces to the molecule, allowing a tight binding between the ligand and receptor.

The structures of indole alkaloids from *Uncaria* were obtained, converted into 3D structures, and saved in.mol2 format using Chemdraw software for molecular docking after energy optimization. Among them, 13 indole alkaloids were successfully docked with sEH (Table 2; Supplementary Table S2), and four alkaloids with the lowest Estimated ΔG were selected for docking result analysis (Figure 4C). Rhynchophylline formed hydrophobic interaction forces with Trp 415 (A), Pro 312 (A), and Val280 (A). Isorhynchophylline (IR) had hydrogen bonding with Asn 414 (A) with an intermolecular distance of 3.27 Å and formed hydrophobic interactions with amino acid residues such as Met 304 (A) and Val 315 (A). Corynoxine (C) formed hydrogen bonds with Pro 305 (A) and Met 304 (A) and hydrophobic interactions with amino acid residues such as Trp 277 (A) and Gln 325 (A). Corynoxine B (CB) had hydrogen bonds with Met304 (A) and hydrophobic interactions with Trp 277 (A), Phe 322 (A), etc. This showed that all four alkaloids could enter the sEH binding pocket and bind stably to the amino acid residues in the pocket. Potential anti-hypertension compounds and sEH inhibitors were discovered through the molecular docking approach.

**TABLE 2:** Binding energies of different alkaloids sEH protein.

Compound	Full Fitness (kcal/mol)	Estimated ΔG (kcal/mol)
CDU	−2564.3965	−8.8276
Uncarine B	−2461.5860	−8.2986
Uncarine A	−2455.6870	−8.3054
Corynoxine B	−2450.8916	−8.6332
Corynoxine	−2449.1235	−9.0312
Rhynchophylline	−2454.3022	−8.8454
Isorhynchophylline	−2446.0030	−8.5065
Corynoxeine	−2443.2860	−8.6532
Isocorynoxeine	−2439.0300	−8.3091
Hirsutine	−2449.8406	−8.0997
Hirsuteine	−2433.6223	−8.0973
Geissoschizine methyl ether	−2457.0188	−7.8905
Isomitraphylline	−2453.3780	−7.6835
Mitraphylline	−2451.8720	−7.5514

The above results indicated that sEH might be the target protein of URE treatment, and potential anti-hypertension ingredients, as well as sEH inhibitors, were discovered through the molecular docking approach. As reported, clinical trials highlighted the promise of sEH inhibitors, such as GSK2256294, a novel sEH inhibitor, attenuated endothelial dysfunction induced by EET decrease on human resistance vessels in a clinical trial of 12 patients with COPD (Yang et al., 2017). On the whole, there was great potential in pursuing sEH as a cardiovascular therapeutic target, and the potential sEH inhibitors in URE were worthy to be deeply explored and verified in future studies.

## 4 Conclusion

In summary, the anti-hypertension effect of URE in the SHR rat model was associated with the reorganization of the perturbed metabolic network, such as the pathway of glycerophospholipid metabolism, linoleic acid metabolism, and arachidonic acid metabolism. Twenty-eight arachidonic acid metabolites in SHR rat model were quantitatively analyzed based on UHPLC-MS method after URE administration for the first time. URE restored the functions of these vasoactive compounds and rebalanced the dynamics of arachidonic acid metabolic flux. Among them, the inhibition of sEH enzyme activity and up-regulation of vasodilators EETs were identified as contributors to the anti-hypertension effect of URE on SHRs. Using the molecular docking approach, thirteen potential anti-hypertension ingredients, as well as sEH inhibitors, were discovered from UR for further validation.

## Data Availability

The datasets presented in this study can be found in online repositories. The names of the repository/repositories and accession number(s) can be found in the article/Supplementary Material.
