# Supplementary material for: Dissecting the Regulation of Arachidonic Acid Metabolites by Uncaria rhynchophylla (Miq). Miq. in Spontaneously Hypertensive Rats and the Predictive Target sEH in the Anti-Hypertensive Effect Based on Metabolomics and Molecular Docking
Source: Front Pharmacol. 2022 May 30;13:909631. doi: 10.3389/fphar.2022.909631 (PMC9196077; doi:10.3389/fphar.2022.909631)
Supplement: Supplementary file 1 [file DataSheet1.docx]

Supplementary Material

# 1. Supplementary Figures and Tables

## Supplementary Figures
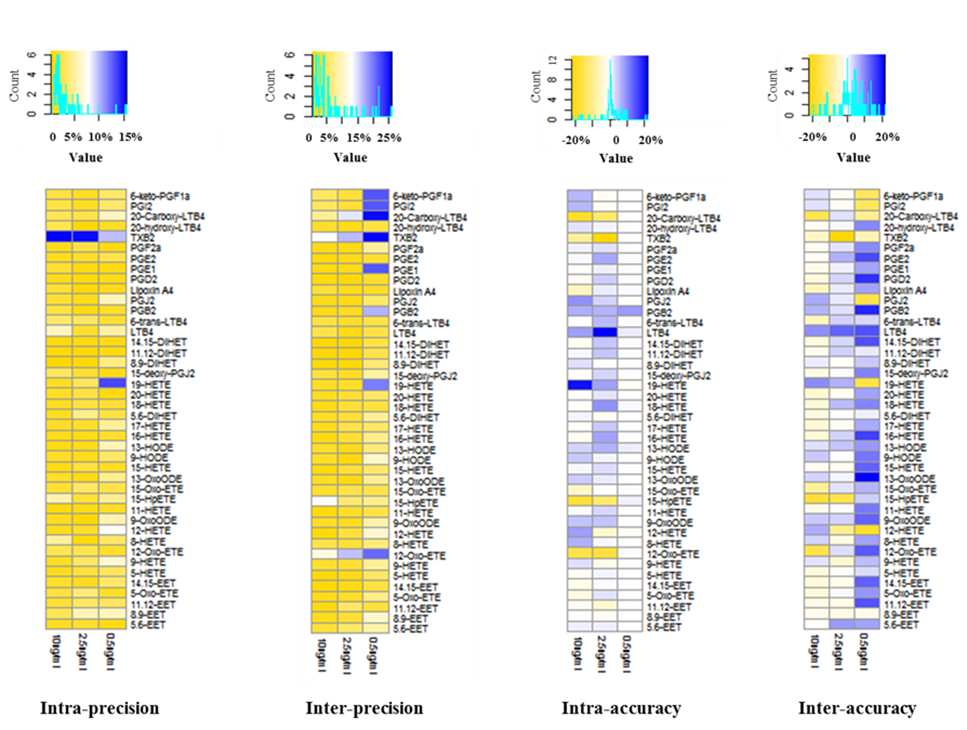


## Supplementary Figure 1. Examination of the precision and accuracy of AA metabolites

##
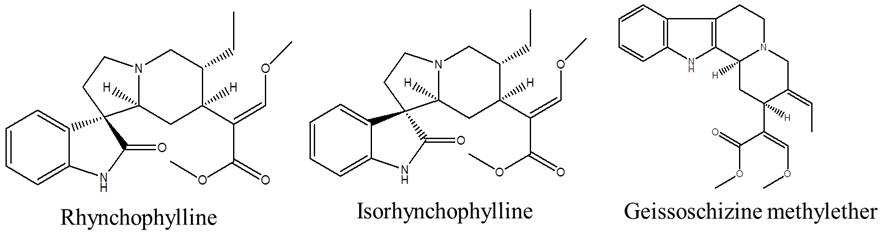


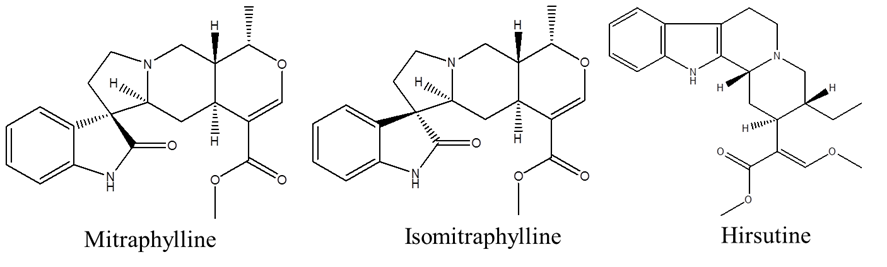

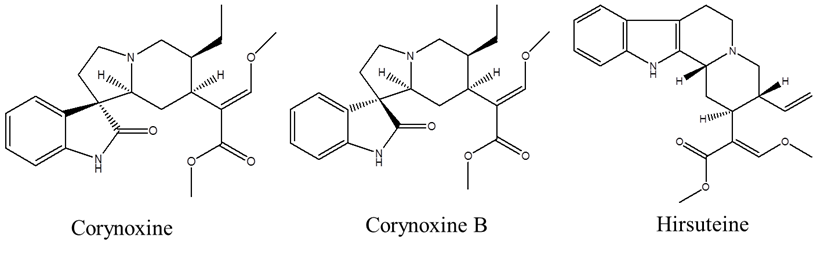

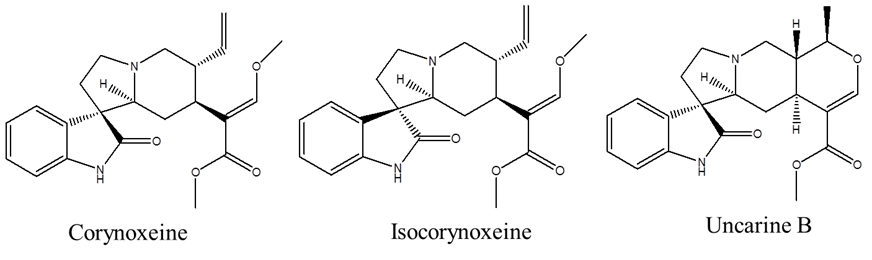


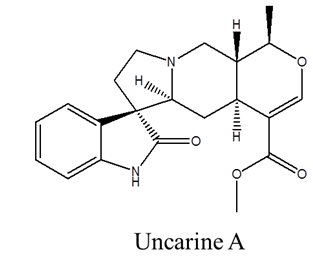


**Supplementary Figure 2.** Structures of 13 alkaloids


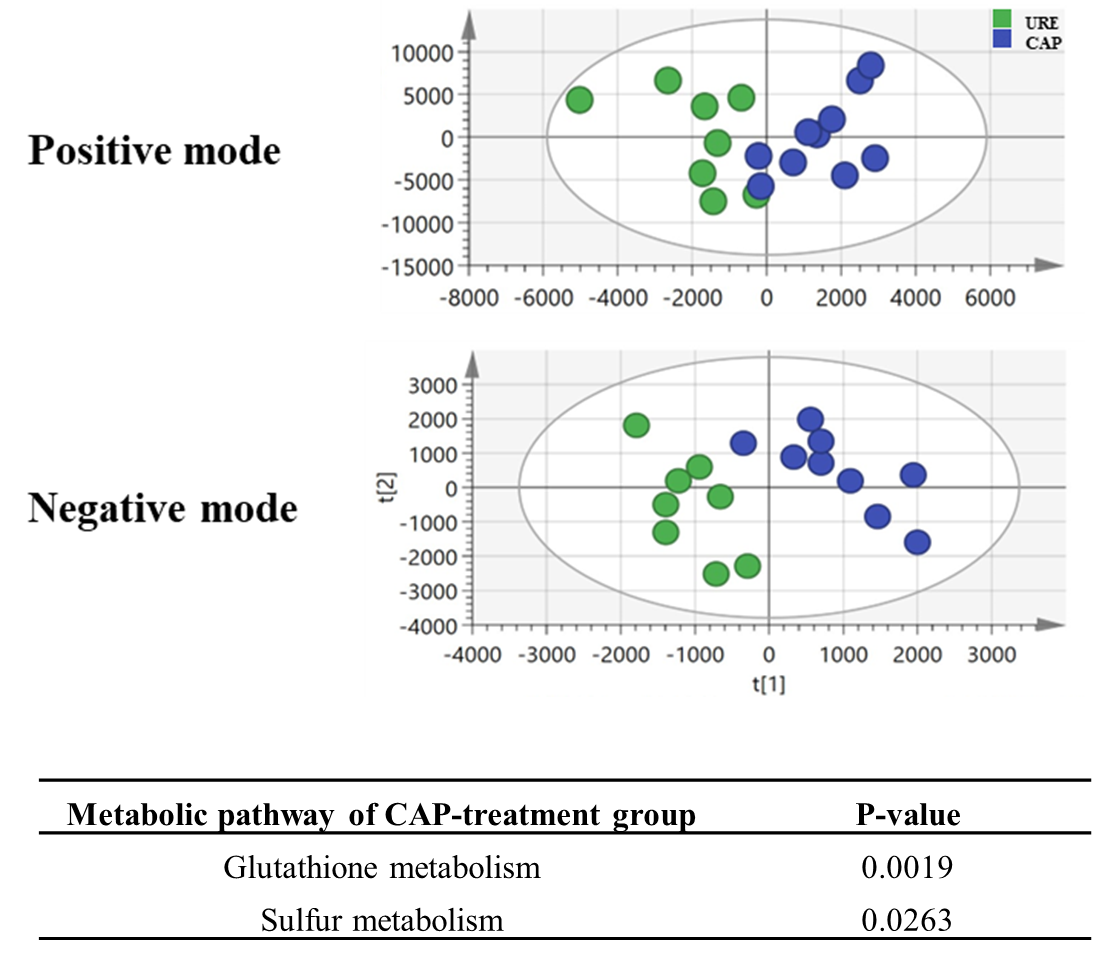


**Supplementary Figure 3.** PCA analysis of URE and CAP metabolomic profiles and the metabolic pathways in CAP-treatment group.

# 1.2 Supplementary Tables

Table S1 sEH hydrophobic interaction of A chain with CDU

| Index | Residue | AA | Distance | Ligand Atom | Protein Atom |
| --- | --- | --- | --- | --- | --- |
| 1 | 337A | VAL | 3.47 | 8194 | 2176 |
| 2 | 372A | VAL | 3.77 | 8200 | 2455 |
| 3 | 381A | TYR | 3.61 | 8186 | 2530 |
| 4 | 406A | PHE | 3.47 | 8183 | 2736 |
| 5 | 524A | TRP | 3.24 | 8183 | 3710 |

Table S2 sEH Hydrogen bonding interaction of A chain with CDU

| Index | Residue | AA | Distance  H-A | Distance  D-A | Donor  Angle | Protein  donor | Sidechain | Donor  Atom |
| --- | --- | --- | --- | --- | --- | --- | --- | --- |
| 1 | 333A | ASP | 2.44 | 3.12 | 125.52 | NO | YES | 8190 [Nam] |
| 2 | 333A | ASP | 2.70 | 3.56 | 146.44 | NO | YES | 8187 [Nam] |
| 3 | 381A | TYR | 2.12 | 3.02 | 160.50 | YES | YES | 2533 [O3] |
| 4 | 382A | GLN | 2.19 | 2.88 | 125.42 | YES | YES | 2542 [Nam] |
| 5 | 465A | TYR | 1.76 | 2.64 | 156.72 | YES | YES | 3218 [O3] |
